# Supplementary material for: Root growth responses to mechanical impedance are regulated by a network of ROS, ethylene and auxin signalling in Arabidopsis
Source: New Phytol. 2021 Feb 10;231(1):225–42. doi: 10.1111/nph.17180 (PMC8651006; doi:10.1111/nph.17180)
Supplement: Supplementary file 1 — Fig. S1 Barrier systems. Fig. S2 proCYCB1;2::CYCB1:2:GUS expression reveals cell division in impeded roots. Fig. S3 Barrier effects on primary root growth. Fig. S4 Analysis of variation in gene expression between and within sample groups. Fig. S5 Genes diﬀerentially expressed in roots encountering a barrier compared with controls after 6 h and 30 h treatment identiﬁed through RNA‐seq. Fig. S6 Treemap output from REVIGO of genes identiﬁed as signiﬁcantly upregulated and downregulated 6 h after encountering a barrier. Fig. S7 Treemap output from REVIGO of genes identiﬁed as signiﬁcantly upregulated and downregulated 30 h after encountering a barrier. Fig. S8 Hormone signalling and metabolic/biosynthesis related GO terms identiﬁed by GO analysis of genes diﬀerentially expressed in response to a barrier. Fig. S9 ABA and GA have no clear role in the root impedance response. Fig. S10 Ethylene‐related gene expression analysis. Fig. S11 Response of etr1 and ein2 to a barrier. Fig. S12 Growth of etr1 between 0 and 8 h after barrier placement. Fig. S13 Growth of wild‐type and ethylene‐sensitive mutant roots after barrier placement. Fig. S14 KEGG pathway mapping of genes diﬀerentially expressed at 6 and 30 h in response to a barrier and identiﬁed as being involved in the auxin signalling pathway. Fig. S15 Confocal imaging of R2D2 in roots responding to a barrier between 0 and 4 h. Fig. S16 Pathways involved in the root barrier response. Table S1 Primers used for qRT‐PCR. Table S2 NADPH‐oxidase genes identiﬁed through RNA‐seq that are upregulated during the barrier response at 6 h. Table S3 List of genes that act as reactive oxygen species (ROS) scavengers identiﬁed in the RNA‐seq data of diﬀerentially expressed genes in response to a barrier log2FC identiﬁed with P‐value < 0.05. Please note: Wiley Blackwell are not responsible for the content or functionality of any Supporting Information supplied by the authors. Any queries (other than missing material) should be directed to [file NPH-231-225-s001.pdf]

New Phytologist Supporting Information

Article title: Root growth responses to mechanical impedance are regulated by a network of ROS, ethylene and auxin signalling in Arabidopsis

Authors: Amy G.R. Jacobsen, George Jervis, Jian Xu, Jennifer F. Topping and Keith Lindsey

Article acceptance date: 5 January, 2021

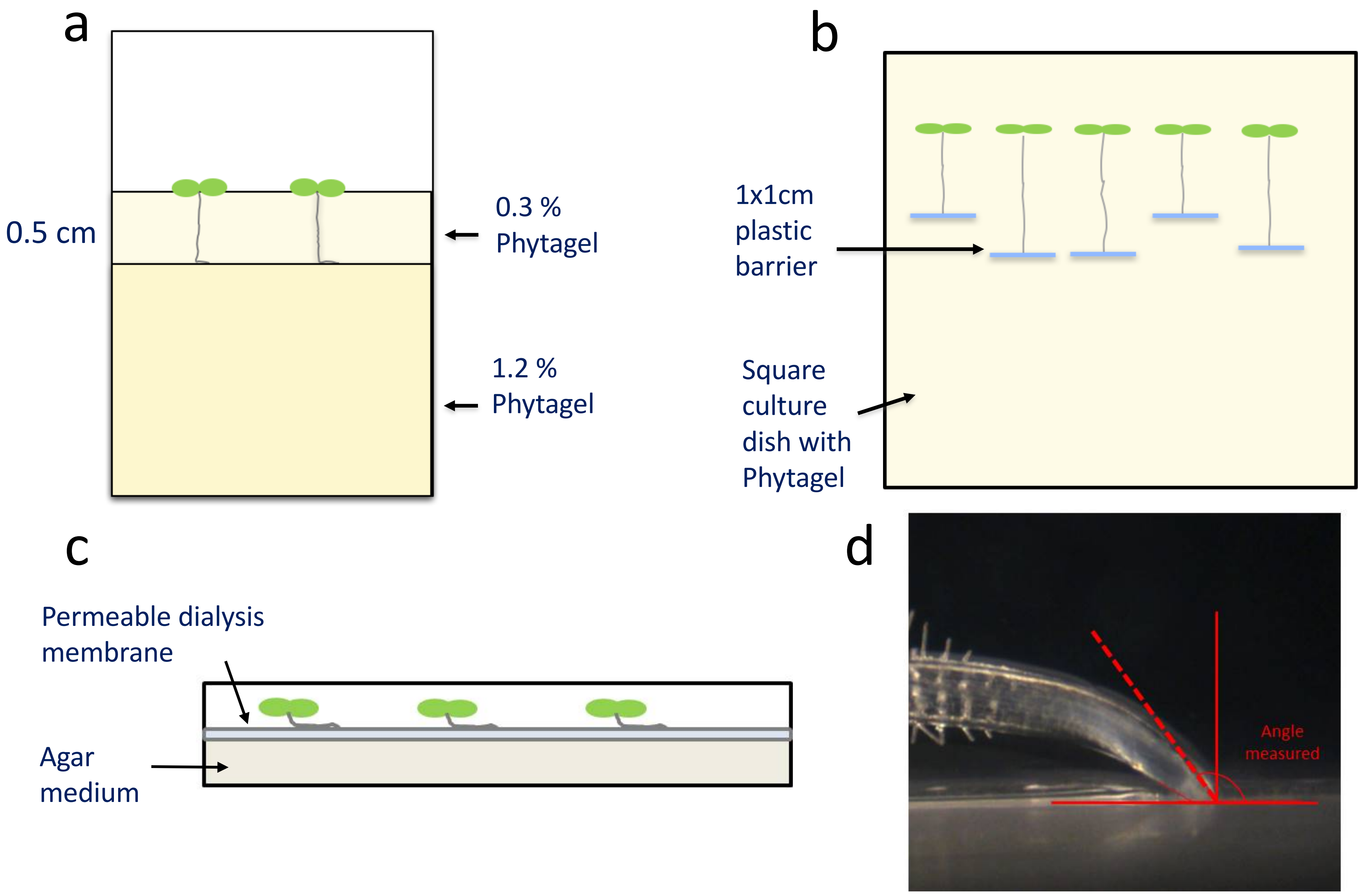

Figure S1. Barrier systems. a) Magenta pot split layer assay. b) Plastic barrier assay. c) Dialysis membrane barrier assay. d) Root tip angle.

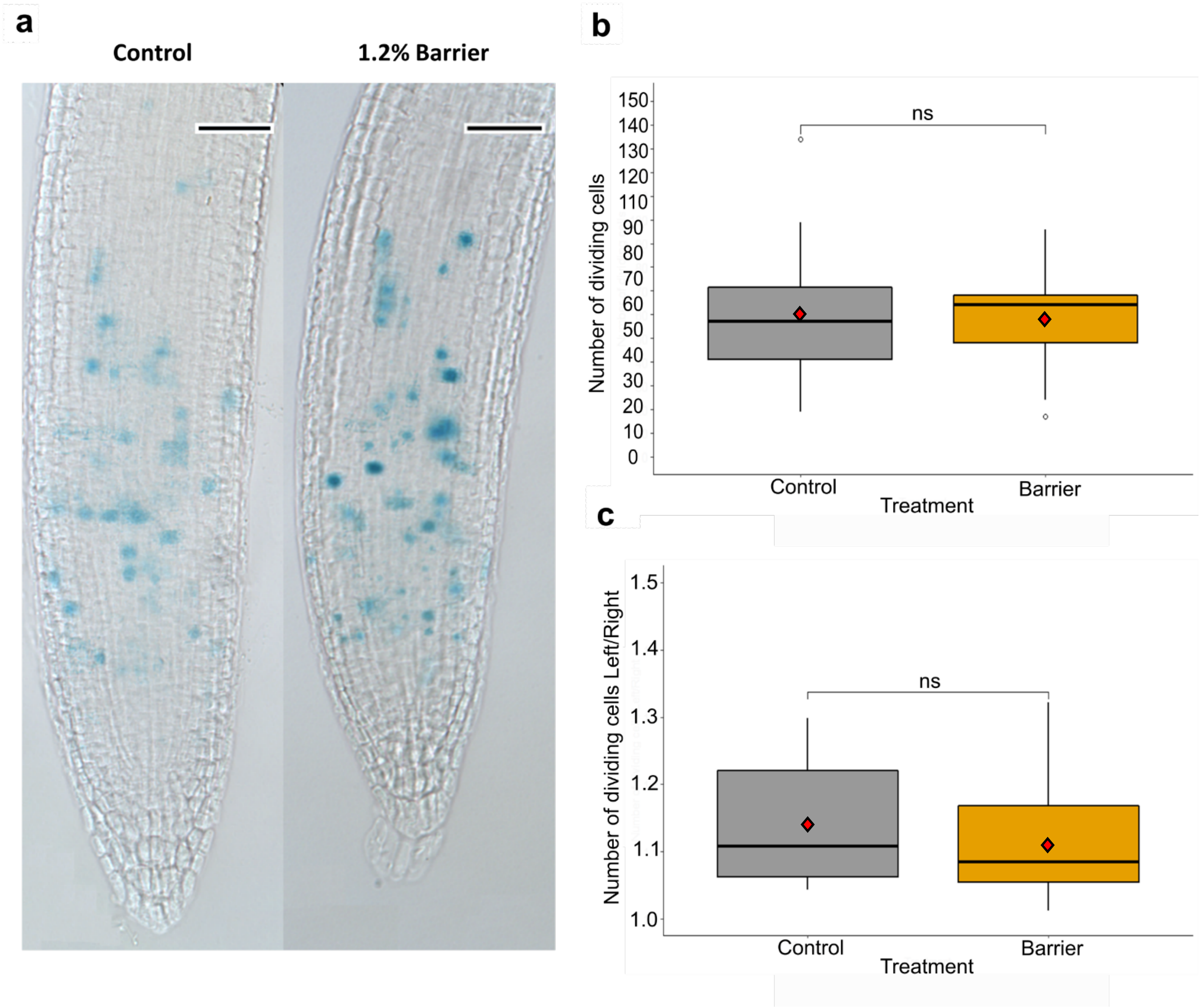

Figure S2. proCYCB1;2::CYCB1:2:GUS expression reveals cell division in impeded roots. a) Typical GUS staining pattern of CYCB1;2:GUS in roots grown in either a single layer of Phytagel (control) or a split layer system consisting of a lower, harder layer to impede growth. b) Number of dividing cells in presence of absence of barrier. c) Ratio of number of dividing cells between the left and right side of the meristem in presence of absence of barrier. Ratio was calculated as exp(|(log(left/right))|). For boxplots, upper and lower boundaries of the box indicate the interquartile range (IQR), a black line within the box marks the median, and whiskers represent the min and max excluding outliers. Open circles represent outliers, and red diamonds represent the mean. Asterisks and brackets indicate significance (ns = not significant). Images and measurement representative of at least 15 samples. Scale bars = 50 µm.

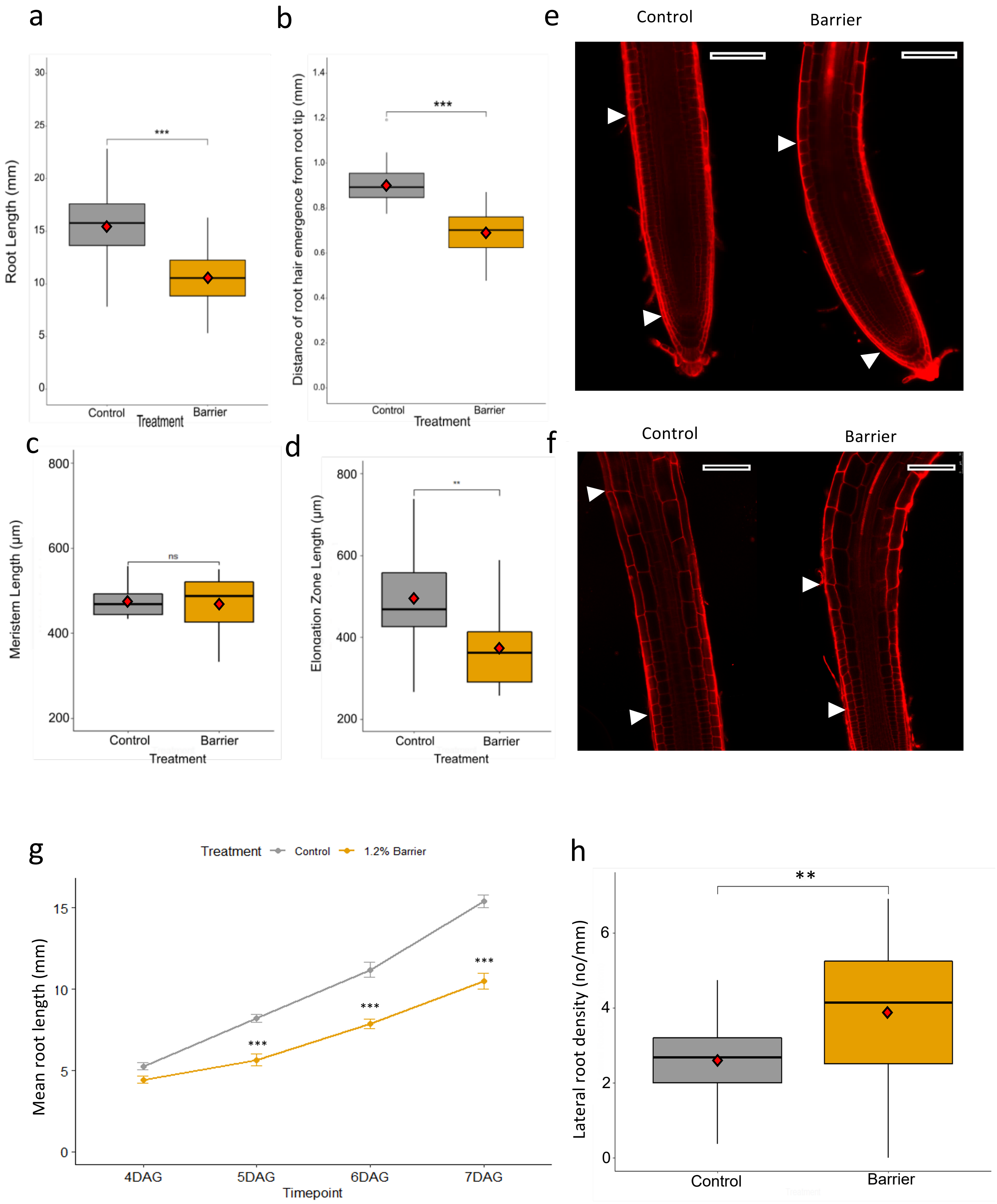

a
Root Length (mm)
***
Control
Barrier
Treatment
b
Distance of root hair emergence from root tip (mm)
***
Control
Barrier
Treatment
e
Control
Barrier
c
Meristem Length (µm)
ns
Control
Barrier
Treatment
d
Elongation Zone Length (µm)
**
Control
Barrier
Treatment
f
Control
Barrier
g
Treatment
Control
1.2% Barrier
Mean root length (mm)
***
4DAG
5DAG
6DAG
7DAG
Timepoint
h
Lateral root density (no/mm)
**
Control
Barrier

Figure S3. Barrier effects on primary root growth. Seeds were sown in a top layer of 0.3% Phytagel and roots grew for 5mm before encountering the lower media layer. a) Root length of 7 DAS seedlings (Student's *t*-test, $P < 0.001$). b) Distance of root hair emergence from the root tip of 7 DAS seedlings (Student's *t*-test, $P < 0.001$). c) Meristem length at 7 DAS (Student's *t*-test, $p = 0.79$). d) Elongation zone length at 7 DAS (Student's *t*-test, $P = 0.003$). e) Primary root tip stained with propidium iodide at 7 DAS. Arrowheads indicate quiescent centre and end of the meristematic zone. f) Elongation zone of primary roots stained with propidium iodide. Arrowheads indicate start and end of the elongation zone. Scale bar = 50 µm. g) Mean primary root length between 4-7 DAS when encountering a barrier. h) Lateral root density (no. of lateral roots per cm primary root) at 11 DAS (t-test, $p = 0.004$).

For boxplots, upper and lower boundaries of the box indicate the interquartile range (IQR), a black line within the box marks the median, and whiskers represent the min and max excluding outliers. Open circles represent outliers, and red diamonds represent means. ns = not significant, **$p < 0.01$, *** $p < 0.001$. Error bars indicate mean ± SE.

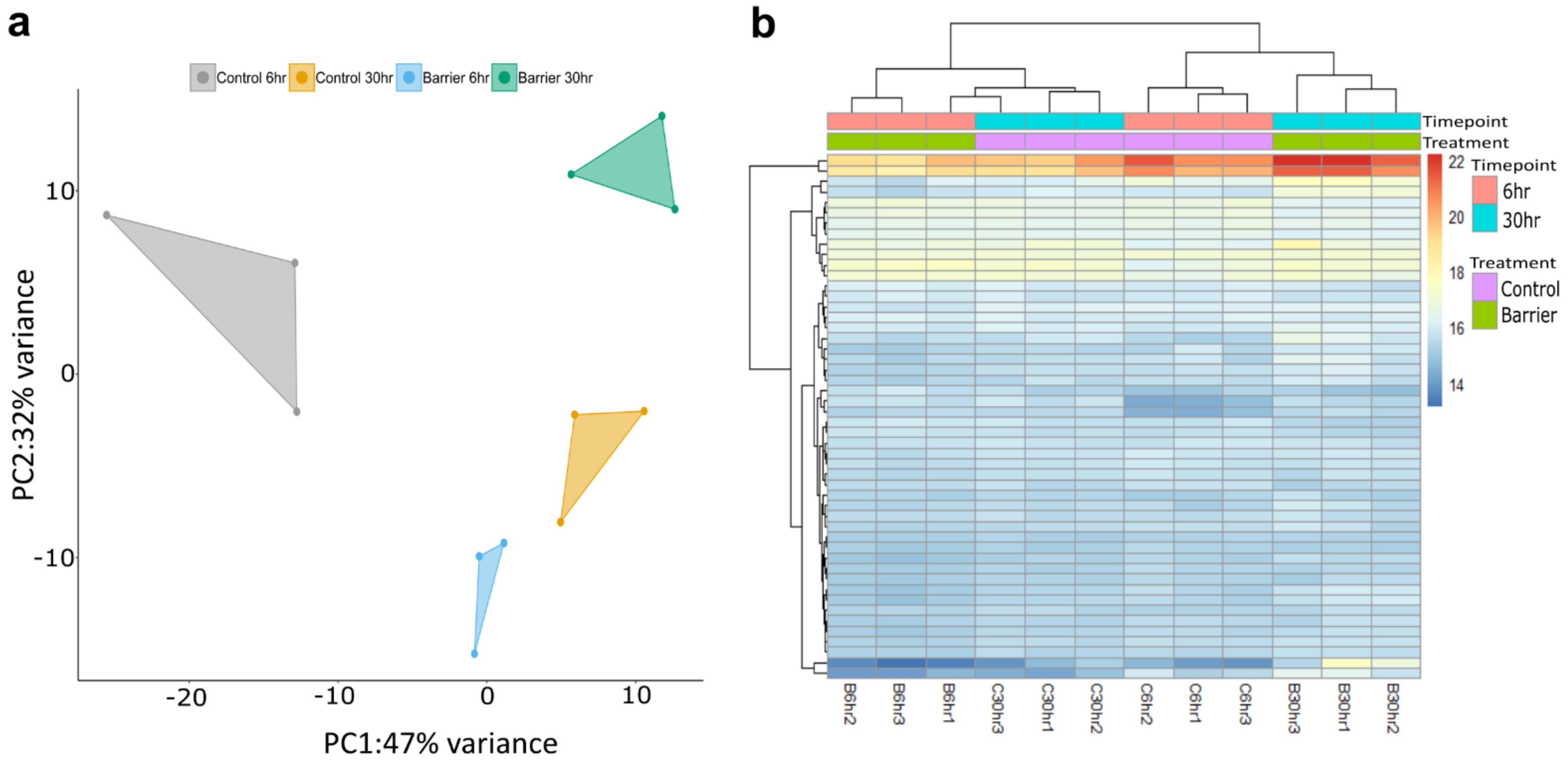

Fig. S4 Analysis of variation in gene expression between and within sample groups. a) Principal component analysis (PCA) plot visualising sample-sample distances. Polygons represent sample groups. PCA performed on regularized logarithm (rlog) transformed data using the R software package DEseq2 . b) Clustered Heatmap of rlog transformed count data for the top 50 most highly expressed genes across all samples. Rows and column aggregated using kmeans clustering by the R package pheatmap.

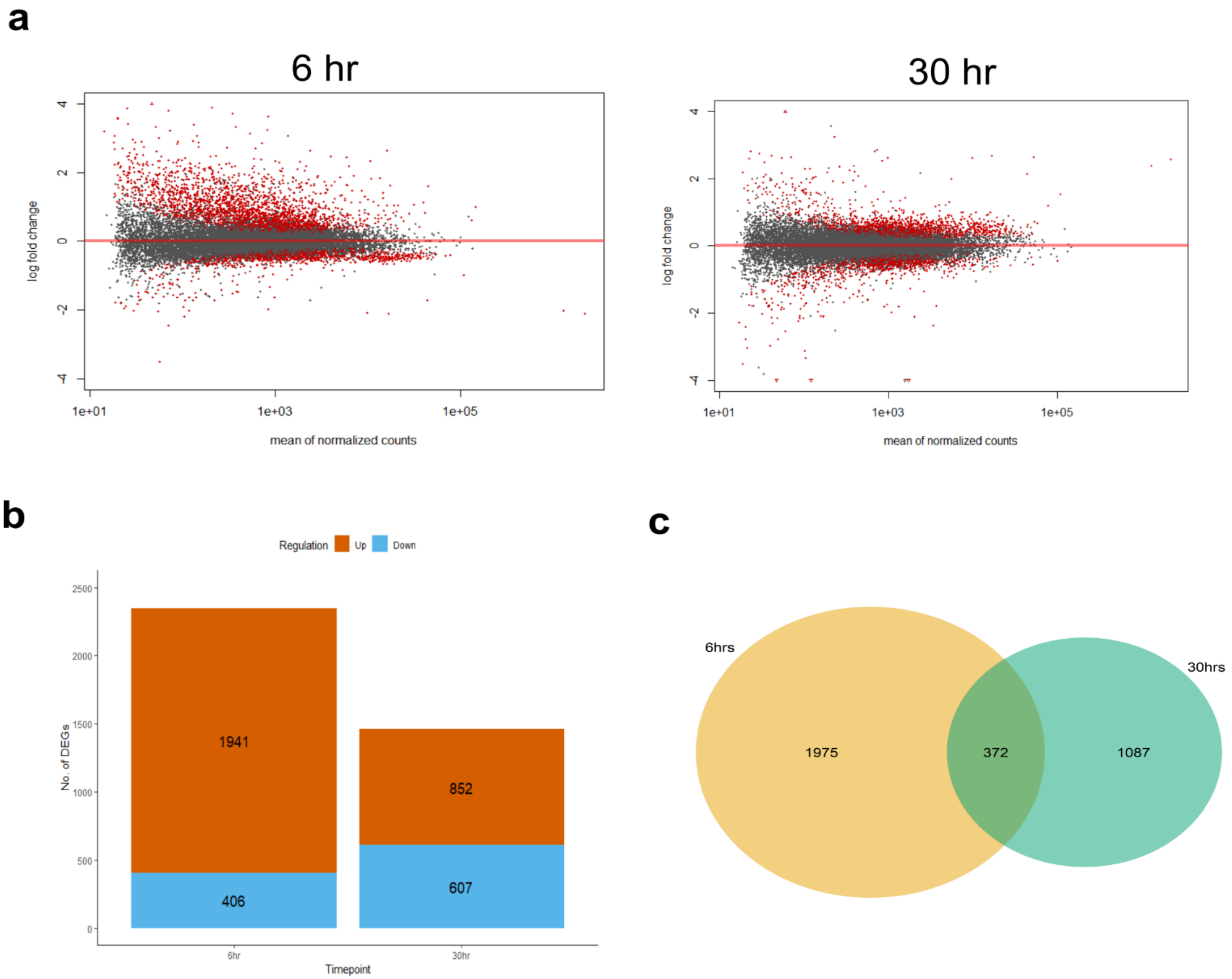

a
6 hr
30 hr
log fold change
mean of normalized counts
1e+01
1e+03
1e+05
b
Regulation
Up
Down
No. of DEGs
1941
406
852
607
6hr
30hr
Timepoint
c
6hrs
30hrs
1975
372
1087

Fig. S5

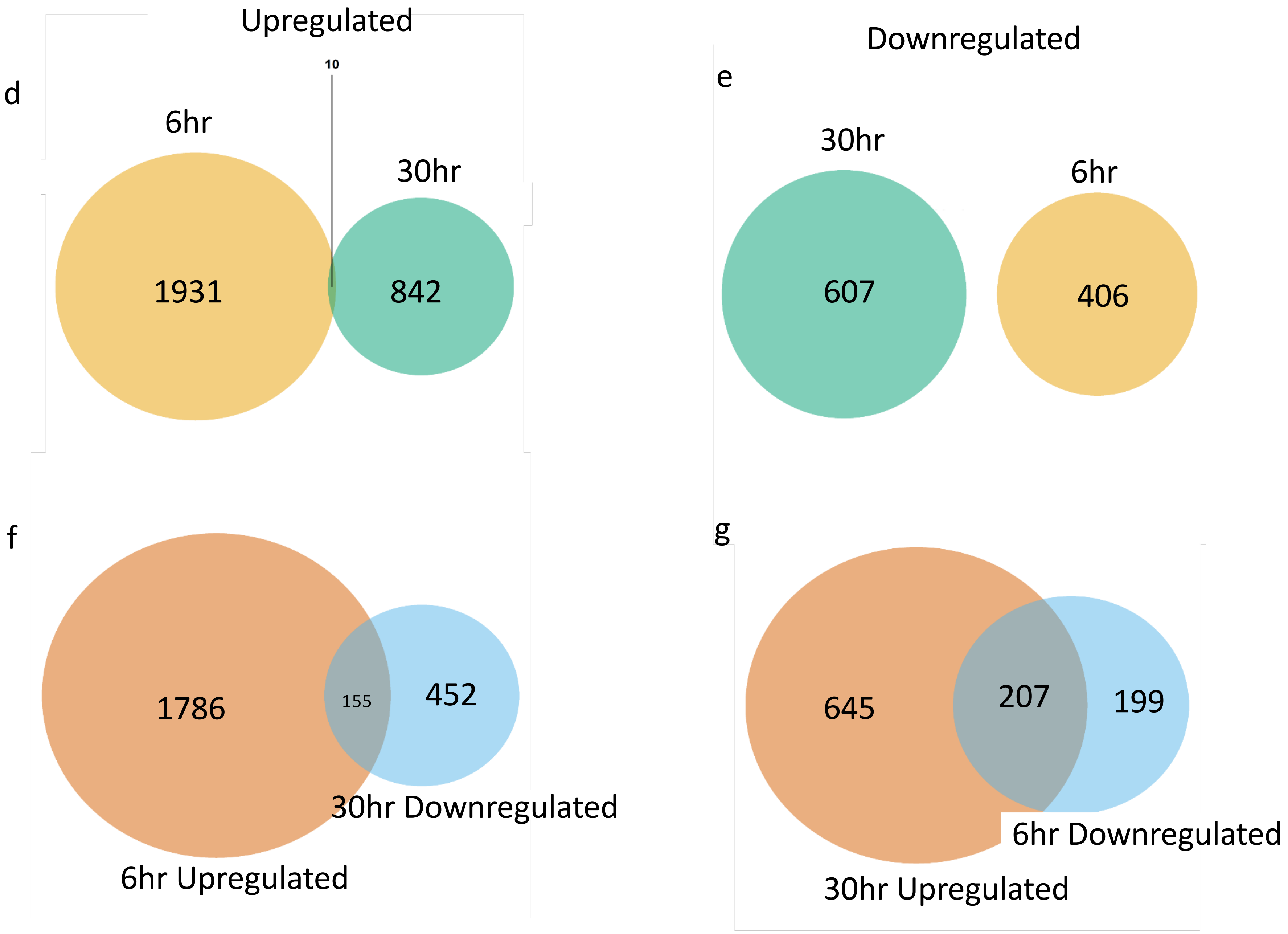

d
Upregulated
10
6hr
30hr
1931
842
e
Downregulated
30hr
6hr
607
406
f
1786
155
452
30hr Downregulated
6hr Upregulated
g
645
207
199
6hr Downregulated
30hr Upregulated

h

6hr Up and 30hr Downregulated Gene Ontology

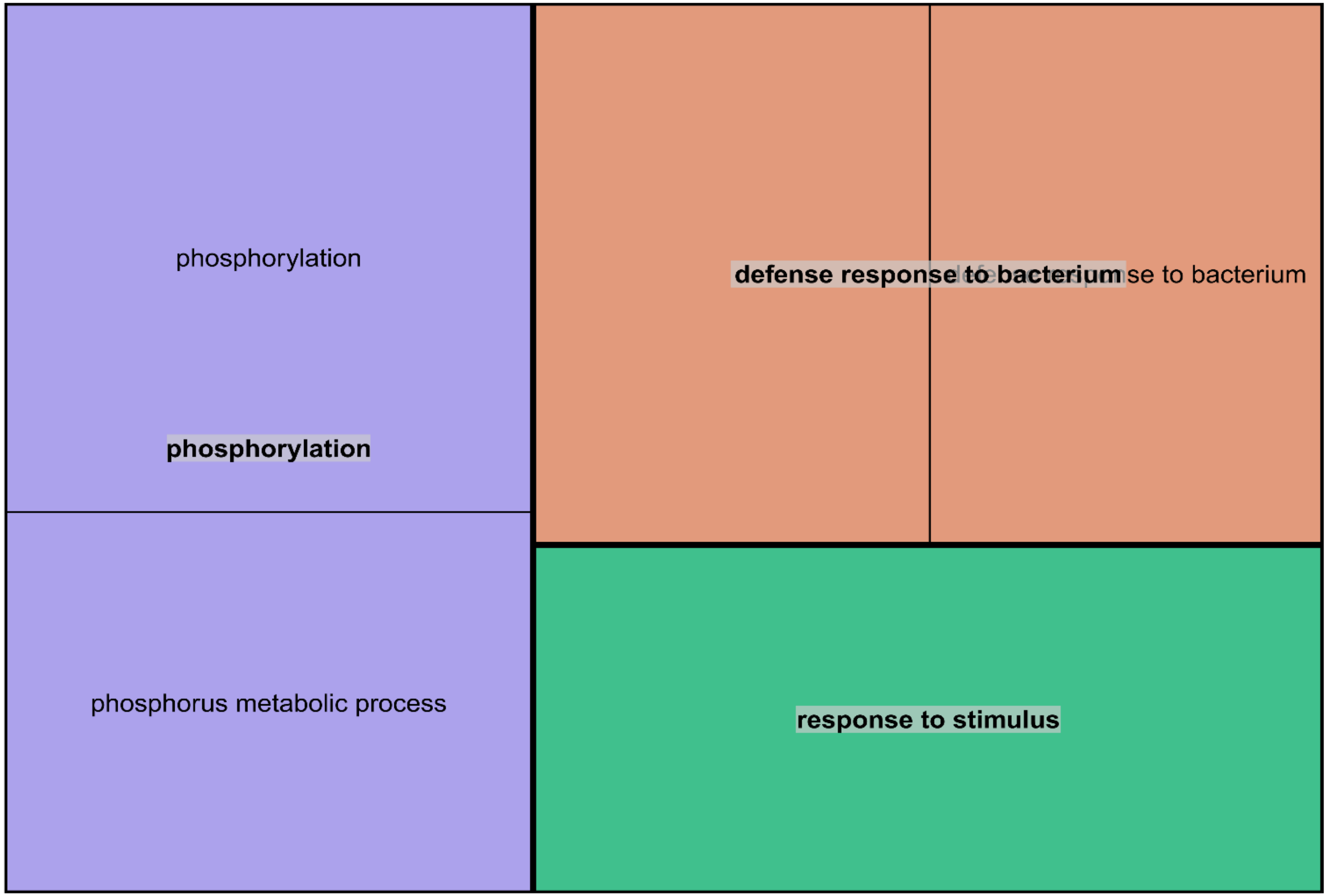

i

6hr Down and 30hr Upregulated Gene Ontology

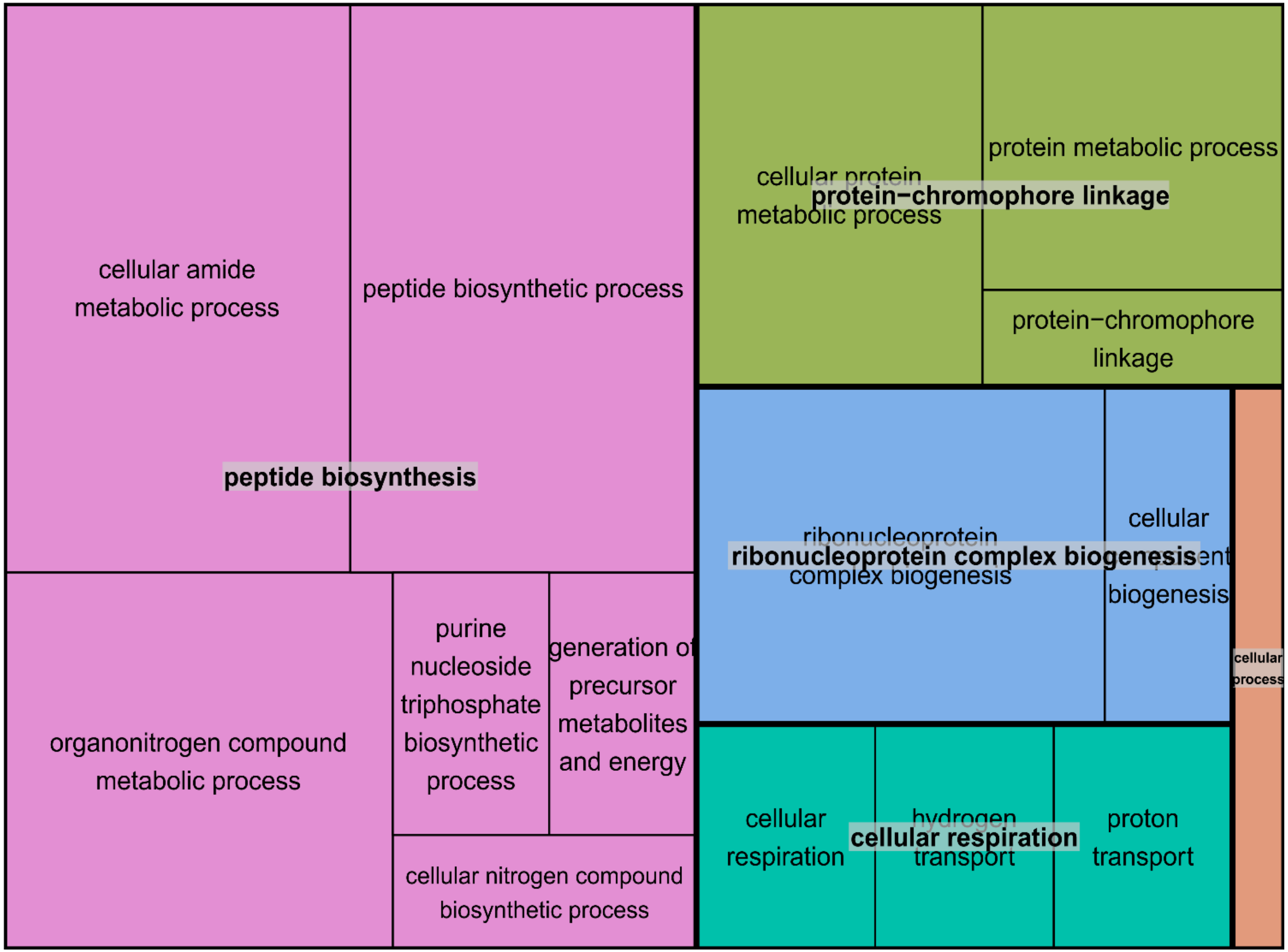

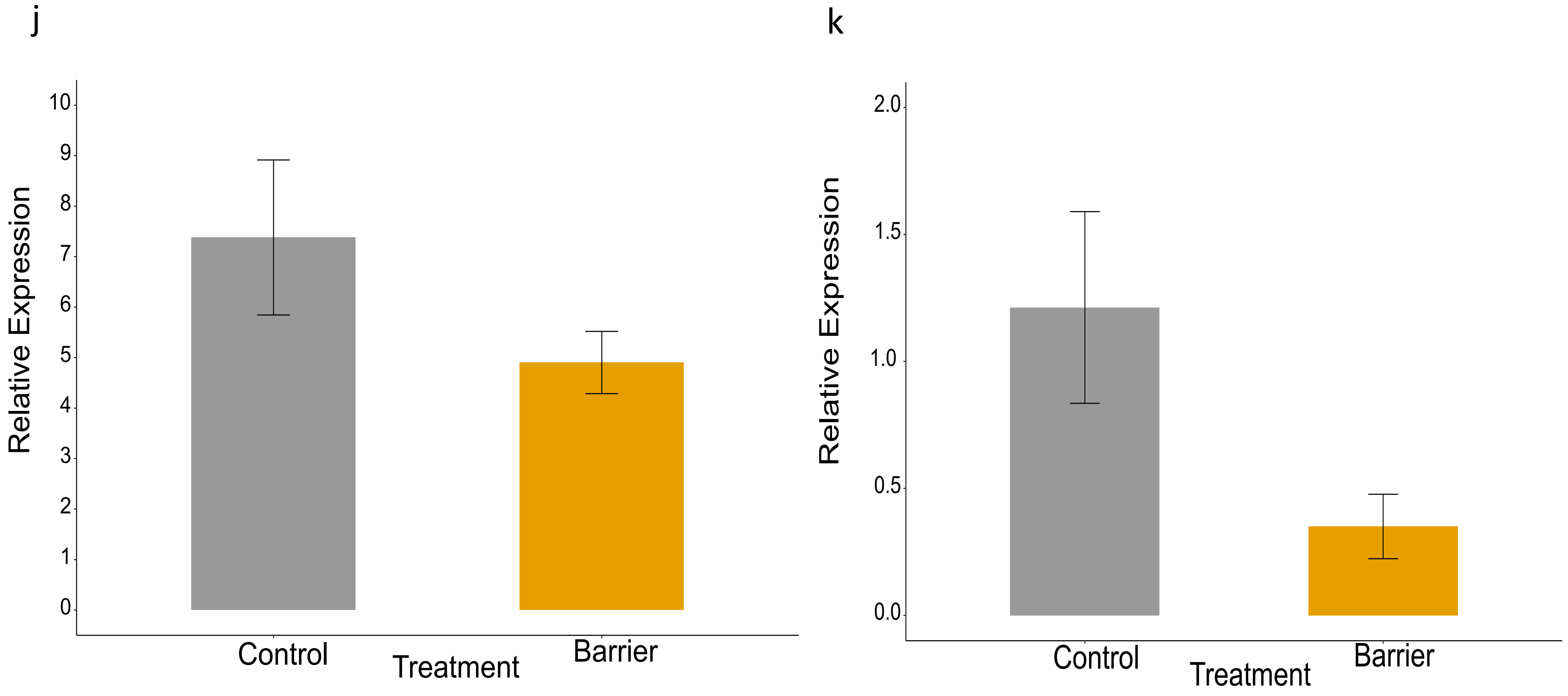
j
k
Relative Expression
10
9
8
7
6
5
4
3
2
1
0
Control
Treatment
Barrier
Relative Expression
2.0
1.5
1.0
0.5
0.0
Control
Treatment
Barrier

Figure S5. Genes differentially expressed in roots encountering a barrier compared with controls after 6 h and 30 h treatment identified through RNA-Seq.
a) Differentially Expressed Genes (DEGs) as estimated by the R software package DEseq2. Red dots represent significant DEGs, black dots non-significant. P-value < 0.05 and a log2 fold change (log2fc) > 0.5 or < - 0.5. b) Significant DEGs separated into up and downregulated genes at the two barrier response timepoints. c) Venn diagram representing overlapping DEGs between treatments. d, e) Venn diagram of DEGs at 6 hours and 30 hours seperated into upregulated genes and downregulated genes. f, g) Venn diagram of DEGs upregulated at 6 h vs genes downregulated at 30 h and genes downregulated at 6 h vs genes upregulated at 30 h. h) Treemap output from REVIGO (Supek et al., 2011) of genes identified as differentially expressed at both 6 h and 30 h after barrier placement. Each rectangle represents a gene ontology (GO) term cluster and each colour represents a supercluster of related clusters. Sizes of rectangles reflect the –log10 P-value of each cluster. i) Treemap output from REVIGO (Supek et al., 2011) of genes identified as differentially expressed at both 6 h and 30 h after barrier placement. Each rectangle represents a gene ontology (GO) term cluster and each colour represents a supercluster of related clusters. Sizes of rectangles reflect the –log10 P-value of each cluster. j, k) Relative normalised counts of *RPL2* 6 h (j) and *IF2/IF5* 30 h (k) after barrier placement, determined by qRT-PCR. Normalised against *PEX4* (AT5G25760). Error bars show mean ± SE.

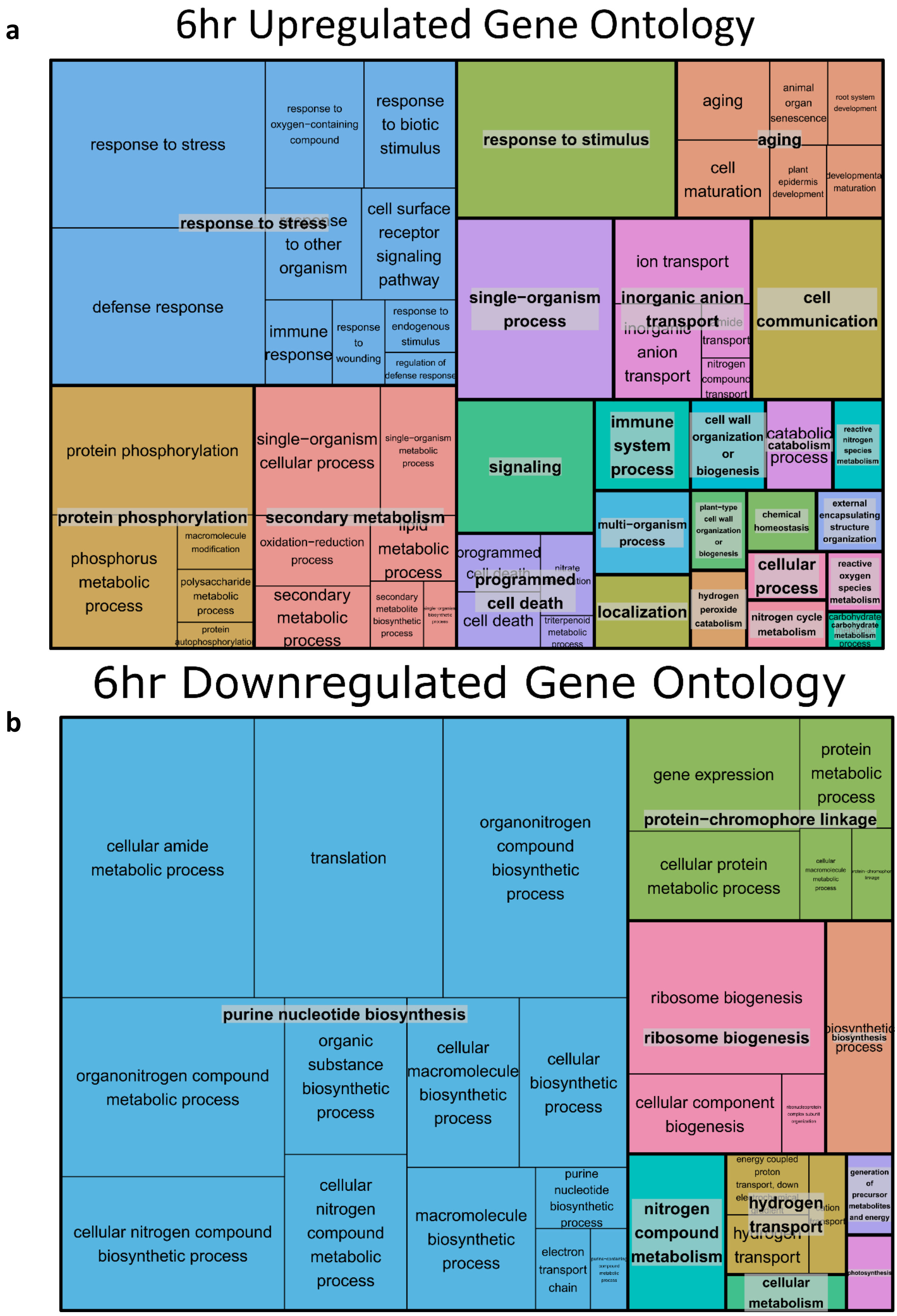

Figure S6. Treemap output from REVIGO (Supek et al., 2011) of genes identified as significantly upregulated **(a)** and downregulated **(b)** 6 hours after encountering a barrier. P-value < 0.05 and a log2 fold change (log2fc) > 0.5 or < -0.5. Each rectangle represents a gene ontology (GO) term cluster and each colour represents a supercluster of related clusters. Sizes of rectangles reflect the –log10 P-value of each cluster.

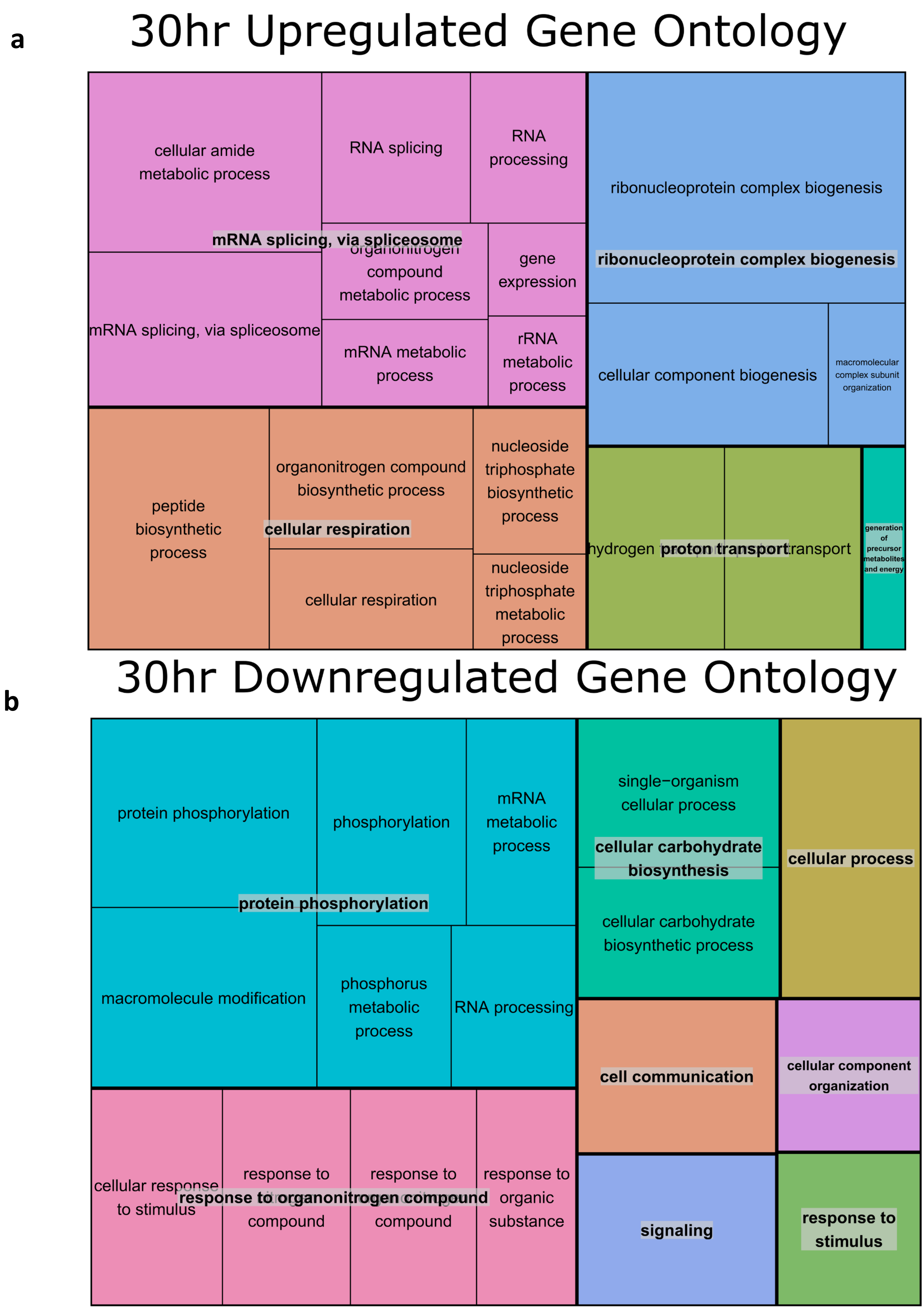

Figure S7. Treemap output from REVIGO (Supek et al., 2011) of genes identified as significantly upregulated **(a)** and downregulated **(b)** 30 h after encountering a barrier. P-value < 0.05 and a log2 fold change (log2fc) > 0.5 or < -0.5. Each rectangle represents a gene ontology (GO) term cluster and each colour represents a supercluster of related clusters. Sizes of rectangles reflect the –log10 P-value of each cluster.

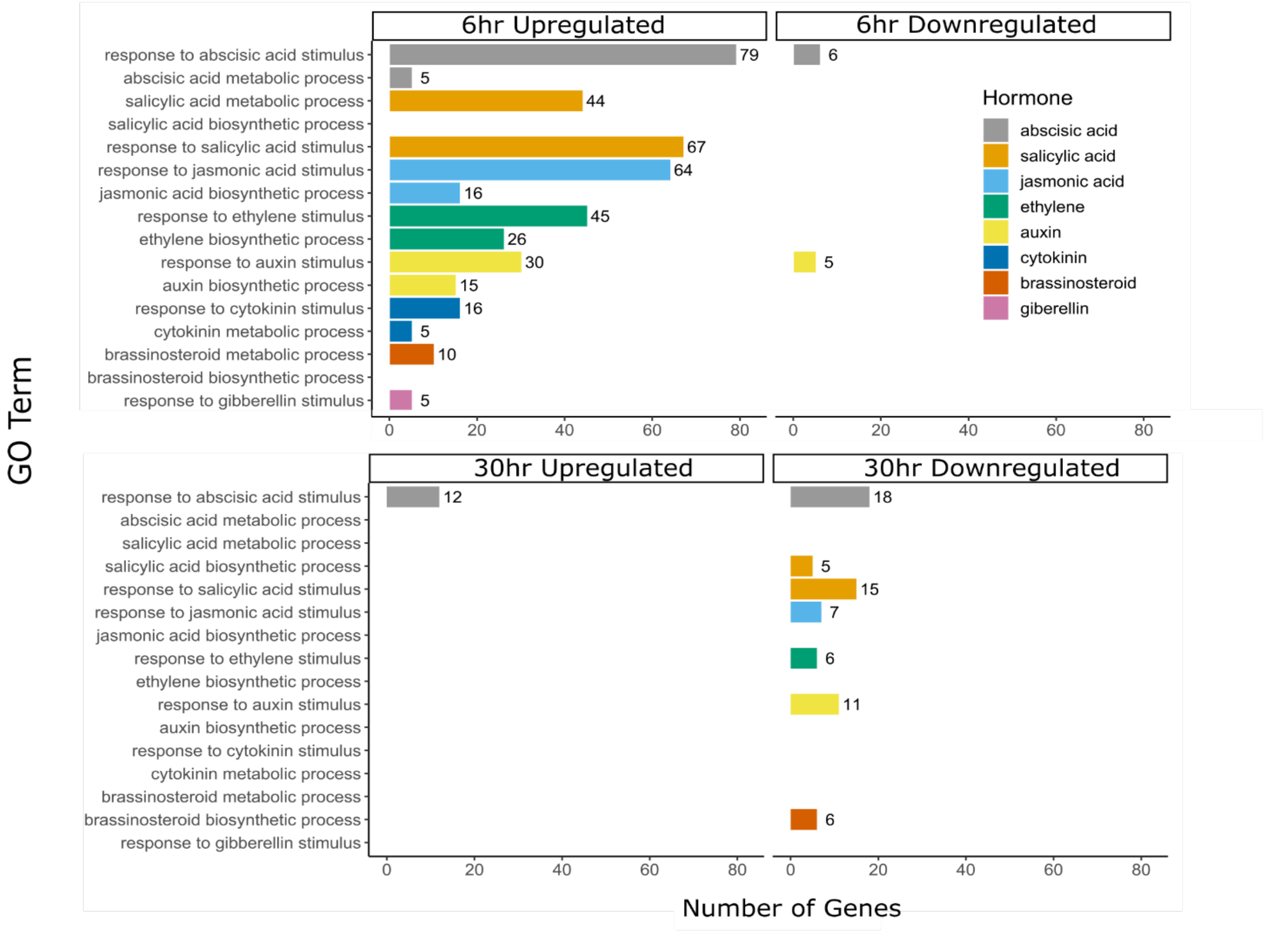

Figure S8. Hormone signalling and metabolic/biosynthesis related GO terms identified by GO analysis of genes differentially expressed in response to a barrier. Bar chart showing numbers of DEGs identified by each GO term. Bars are grouped by the hormone the GO term relates to.

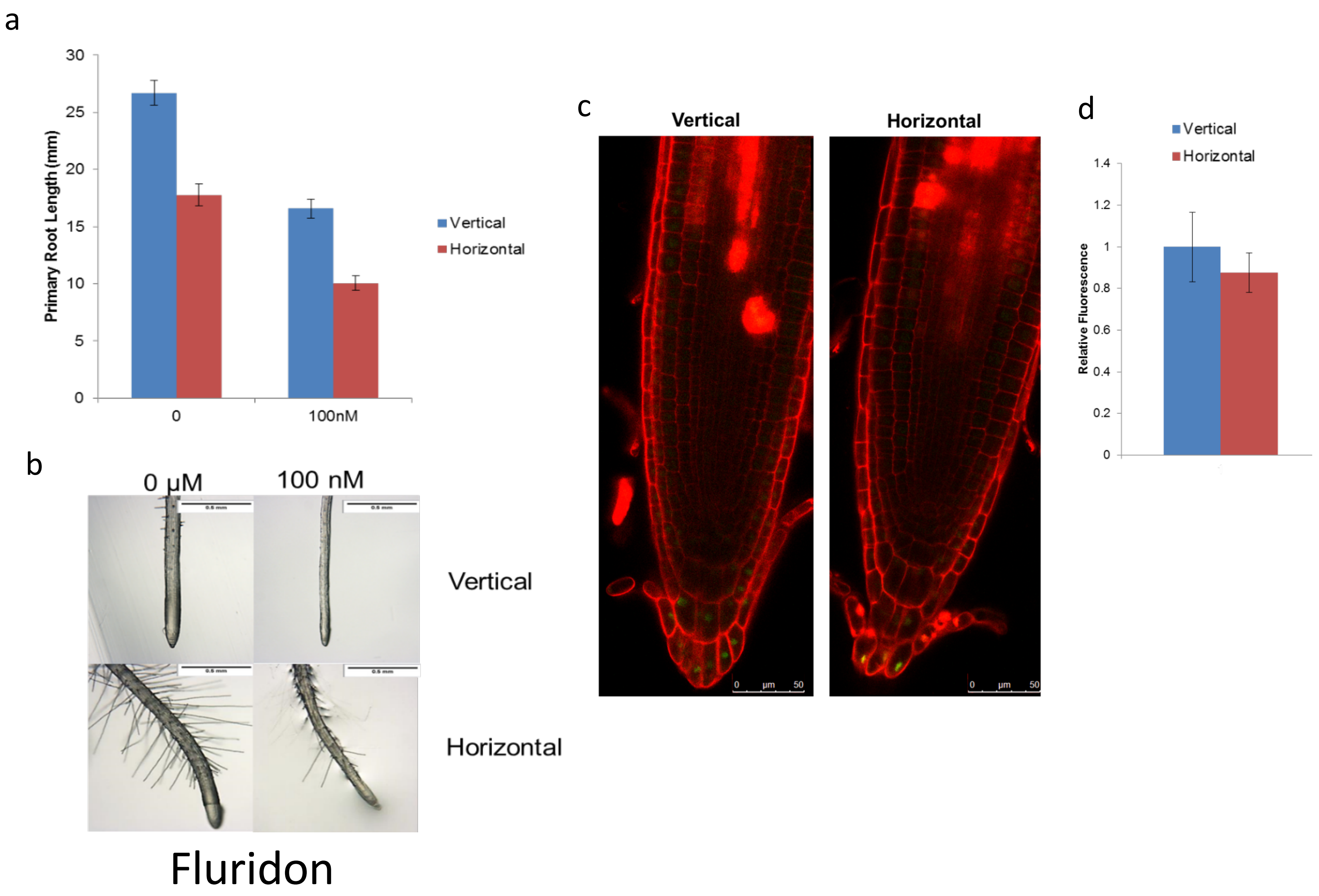
a
Primary Root Length (mm)
30
25
20
15
10
5
0
0
100nM
Vertical
Horizontal
b
0 µM
100 nM
Vertical
Horizontal
Fluridon
c
Vertical
Horizontal
0 µm 50
0 µm 50
d
Vertical
Horizontal
Relative Fluorescence
1.4
1.2
1
0.8
0.6
0.4
0.2
0

Figure S9. ABA and GA have no clear role in the root impedance response.
a, b. Effects of the ABA synthesis inhibitor fluoridon (100 nM) on (a) primary root length and (b) phenotype of roots grown without (vertical) or with (horizontal) a horizontal mechanical barrier. No relative change in root growth was seen in fluoridon and control roots on contact with a dialysis membrane barrier at 7 DAS. Scale bars in (b) = 0.8 mm.

c, Laser scanning confocal image of *Arabidopsis* root expressing RGA:GFP. Green: RGA:GFP Red: propidium iodide stain. Scale bar indicates 50 µm. d, Relative expression of RGA:GFP in columella cells of the root tip of 7d old *Arabidopsis* seedlings. Seedlings were grown in the presence of a dialysis membrane and imaged at 7 DAG. Representative of 6-8 images per treatment.

(a,d): Error bars represent mean ± SE,

a

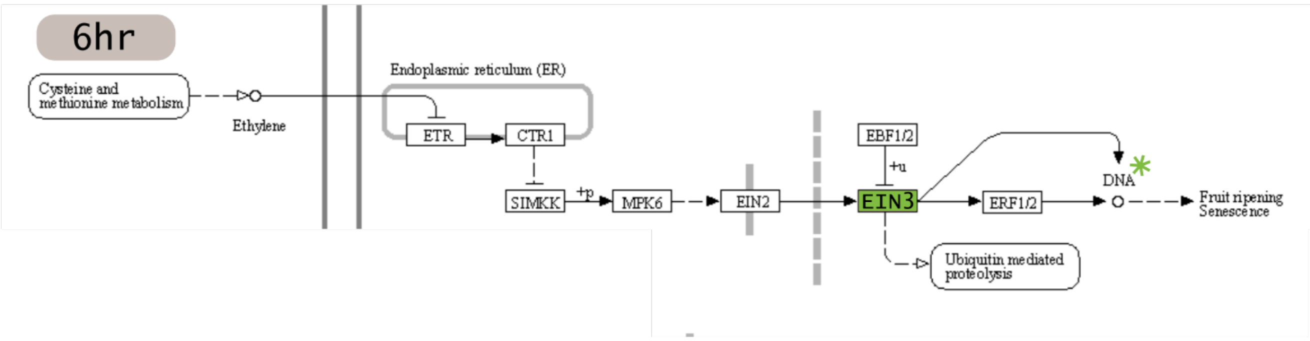

Locus ID	Gene Symbol	LogFC	KEGG Orthology ID
AT5G21120	EIL2	2.092	ethylene-insensitive protein 3 (EIN3)

Locus ID	Gene Symbol	LogFC	Gene description
AT4G17500	ERF-1 *	0.959	Encodes a member of the ERF (ethylene response factor) subfamily B-3 of ERF/AP2 transcription factor family (ATERF-1).
AT3G15210	ERF4 *	0.769	Encodes a member of the ERF (ethylene response factor) subfamily B-1 of ERF/AP2 transcription factor family (ATERF-4).

b

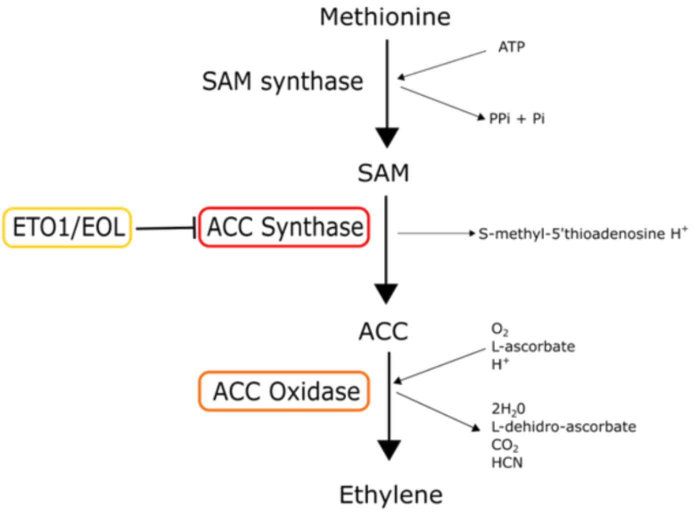

Locus ID	Gene Symbol	LogFC 6hr	LogFc 30hr	Gene Description
AT5G65800	ACC SYNTHASE 5 (ACS5)	2.520	-1.02	1-aminocyclopropane-1-carboxylate synthase (ACS) is encoded by a multigene family consisting of at least five members whose expression is induced by hormones, developmental signals, and protein synthesis inhibition
AT1G05010	ETHYLENE-FORMING ENZYME (EFE)	0.794	0	Encodes 1-aminocyclopropane-1-carboxylate oxidase
AT5G58550	ETO1-LIKE 2 (EOL2)	0.697	-1.5	Encodes a paralog of ETO1, which is a negative regulator of ACS5 (a key enzyme in ethylene biosynthesis pathway). EOL2 also interacts with and inhibits the activity of ACS5

Figure S10. Ethylene-related gene expression analysis. a) KEGG Pathway mapping of genes differentially expressed at 6 h in response to a barrier and identified as being involved in the ethylene signalling pathway. Genes present within the data set are highlighted with colours corresponding to KEGG Orthology (molecular function) definition. b) Genes differentially expressed at 6 h in response to a barrier and identified as being involved in the ethylene biosynthesis pathway. Components of the pathway with an identified DEG are highlighted red (ACC Synthase), orange (ACC Oxidase) and yellow (ETO1/EOL1).

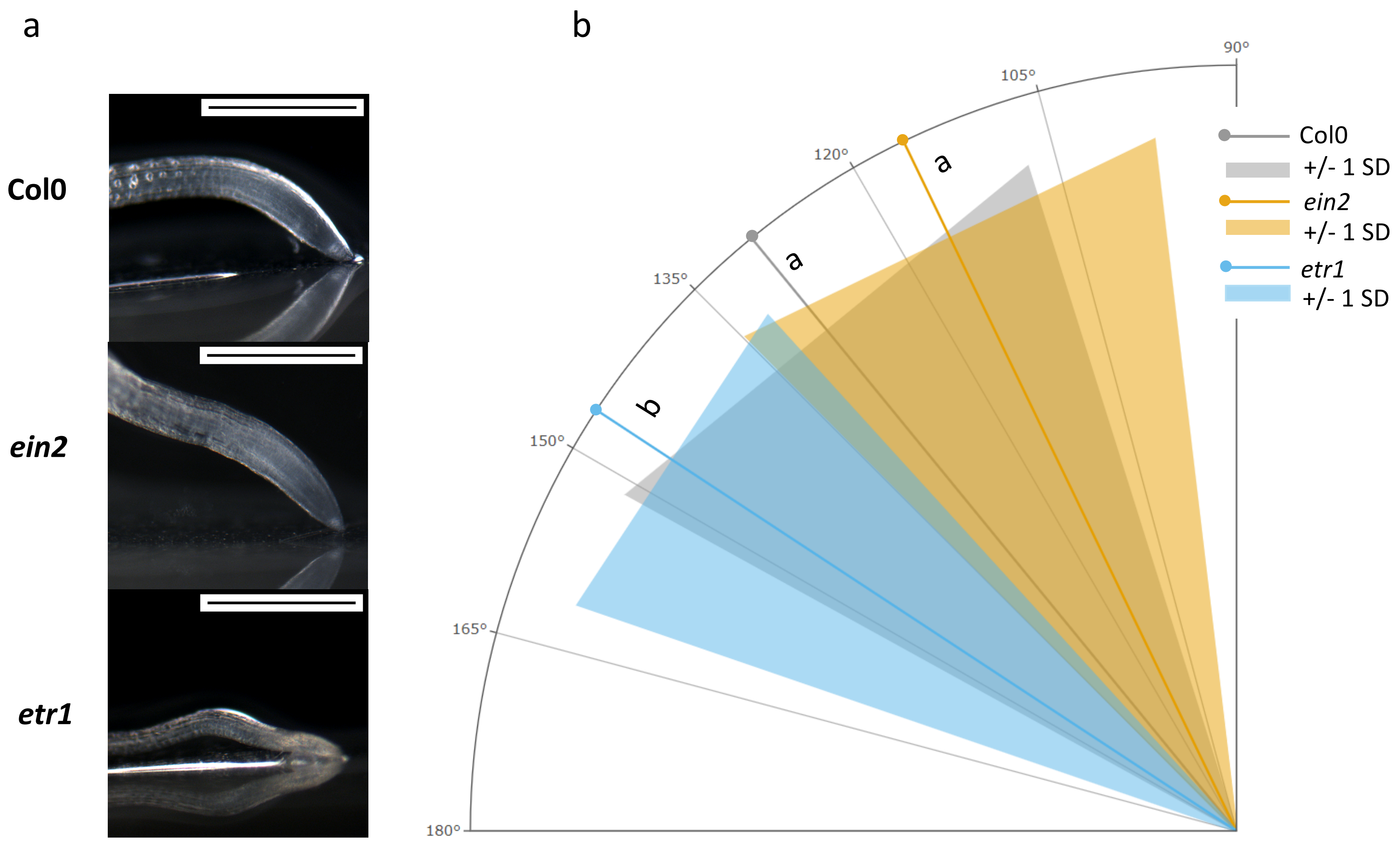

Figure S11. Response of *etr1* and *ein2* to a barrier. a) Plastic barriers were placed in front of primary roots of seedlings 6 DAS and root tips were imaged at 24 h after encountering a barrier. Scale bar indicates 0.5 mm. b) Angle of primary root tips to the horizontal barrier. Lines indicate mean and surrounding shaded area indicates ± SD. Letters indicate significance with a Tukey Pairwise comparison $P < 0.05$

a

b

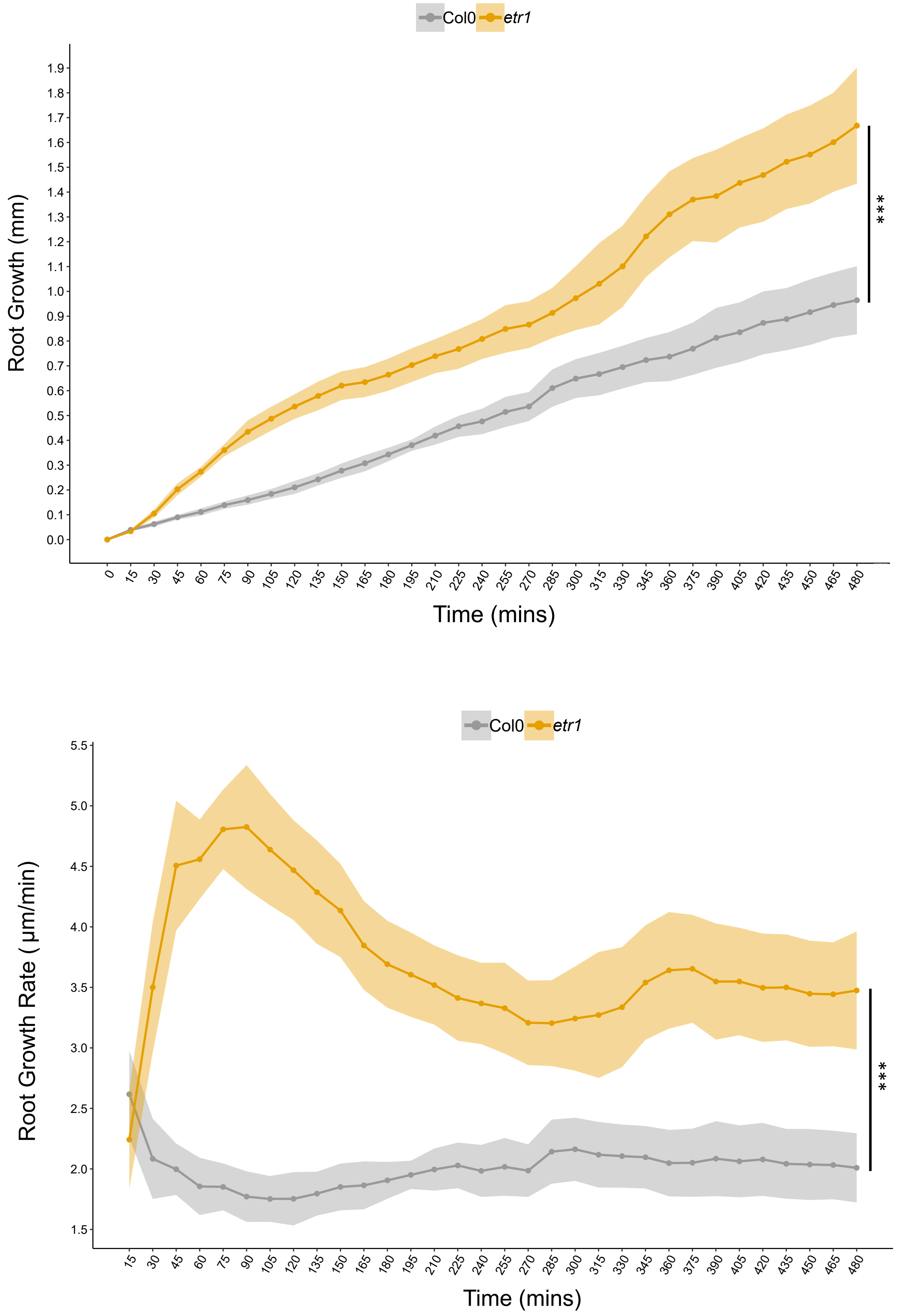

Figure S12. Growth of *etr1* between 0 and 8 hours after barrier placement. Root growth as measured by time-lapse imaging of roots encountering a barrier. a) Root growth of the primary root tip between 0-480 minutes after contact with the barrier. b) Root growth rate (µm/min) between 15-480 minutes after encountering a barrier. Plastic barriers were placed in front of 6 day old vertically growing roots and root tips were imaged every 15 min. Lines and dots indicate mean with shaded area indicating ± SE. Letters indicate significance after a *Student's* t-test (*** < 0.001).

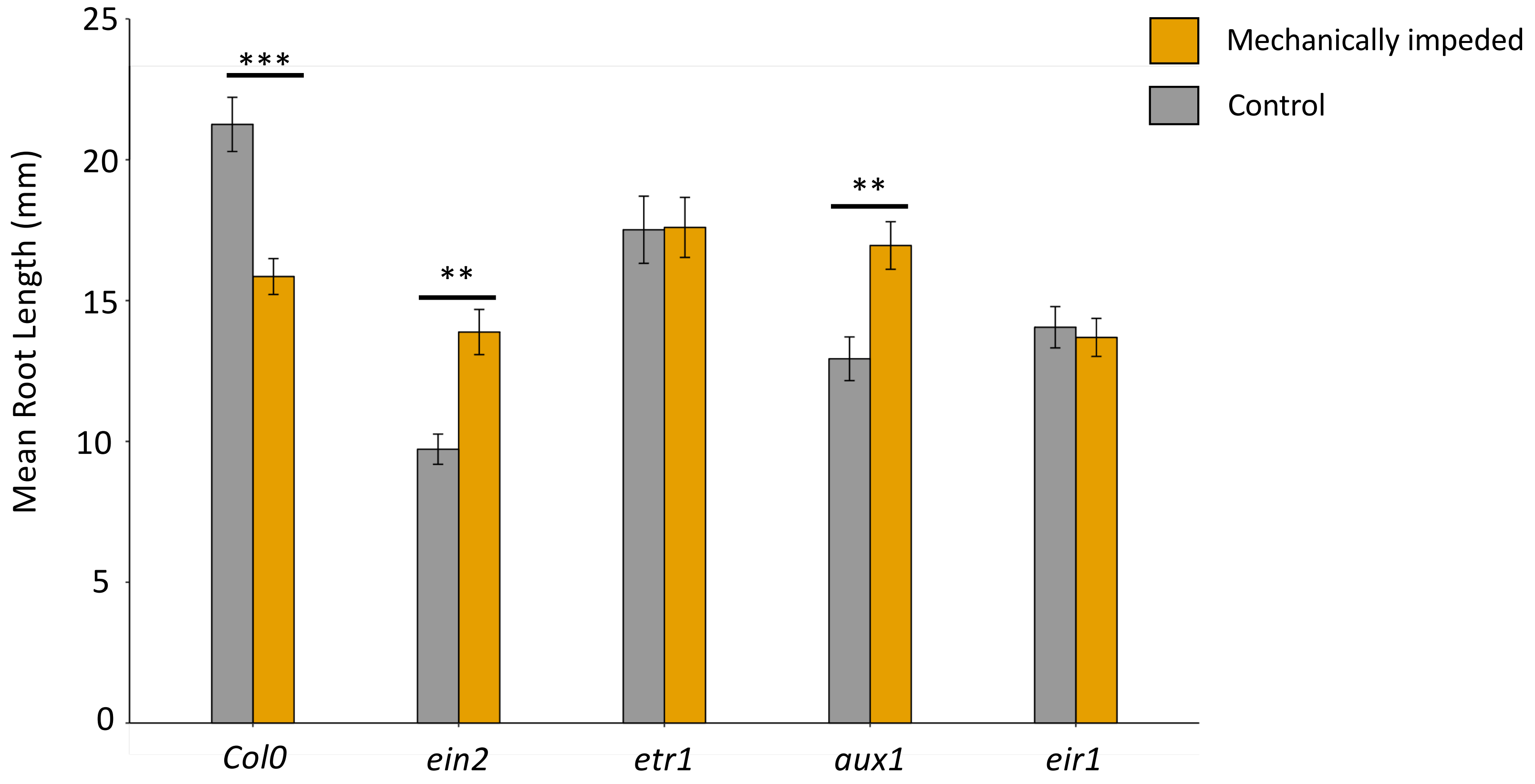

Figure S13. Growth of wildtype and ethylene-sensitive mutant roots after barrier placement. Primary root length was measured after growth of seedlings on dialysis membrane barriers for 7 DAS. Error bars represent standard error of the mean of 15 seedlings per treatment. Root growth of the mutants was not significantly inhibited by barrier impedance (ANOVA and Tukey pairwise comparison, *** = P <0.0001, ** + P <0.001).

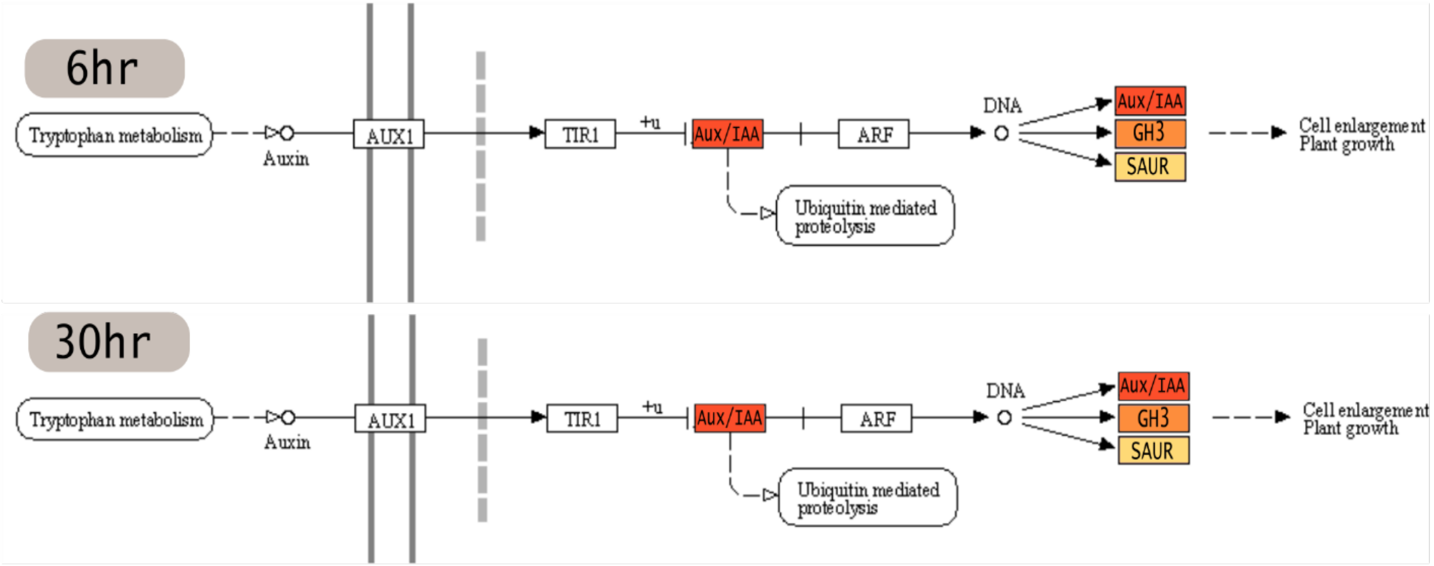

6hr

Locus ID	Gene Symbol	LogFC	KEGG Orthology ID
AT4G29080	PAP2	-0.58	auxin-responsive protein IAA **(AUX/IAA)**
AT1G2830	GH3.17	1.18	auxin-responsive GH3 gene family **(GH3)**
AT4G37390	BRU6	1.19	auxin-responsive GH3 gene family **(GH3)**
AT5G54510	DFL1	0.65	auxin-responsive GH3 gene family **(GH3)**
AT2G45210	SAUR36	-0.77	SAUR family protein **(SAUR)**
AT5G50760	SAUR55	0.65	SAUR family protein **(SAUR)**

30hr

Locus ID	Gene Symbol	LogFC	KEGG Orthology ID
AT4G29080	IAA30	-0.58	auxin-responsive protein IAA **(AUX/IAA)**
AT1G2830	IAA14	1.18	auxin-responsive protein IAA **(AUX/IAA)**
AT4G37390	PAP2	1.19	auxin-responsive protein IAA **(AUX/IAA)**
AT5G54510	GH3.17	0.65	auxin-responsive GH3 gene family **(GH3)**
AT2G45210	DFL2	-0.77	auxin-responsive GH3 gene family **(GH3)**
AT5G50760	SAUR36	0.65	SAUR family protein **(SAUR)**

Figure S14. KEGG Pathway mapping of genes differentially expressed at 6 and 30 hours in response to a barrier and identified as being involved in the auxin signalling pathway. Genes present within the data set are highlighted with colours corresponding to KEGG Orthology (molecular function) definition.

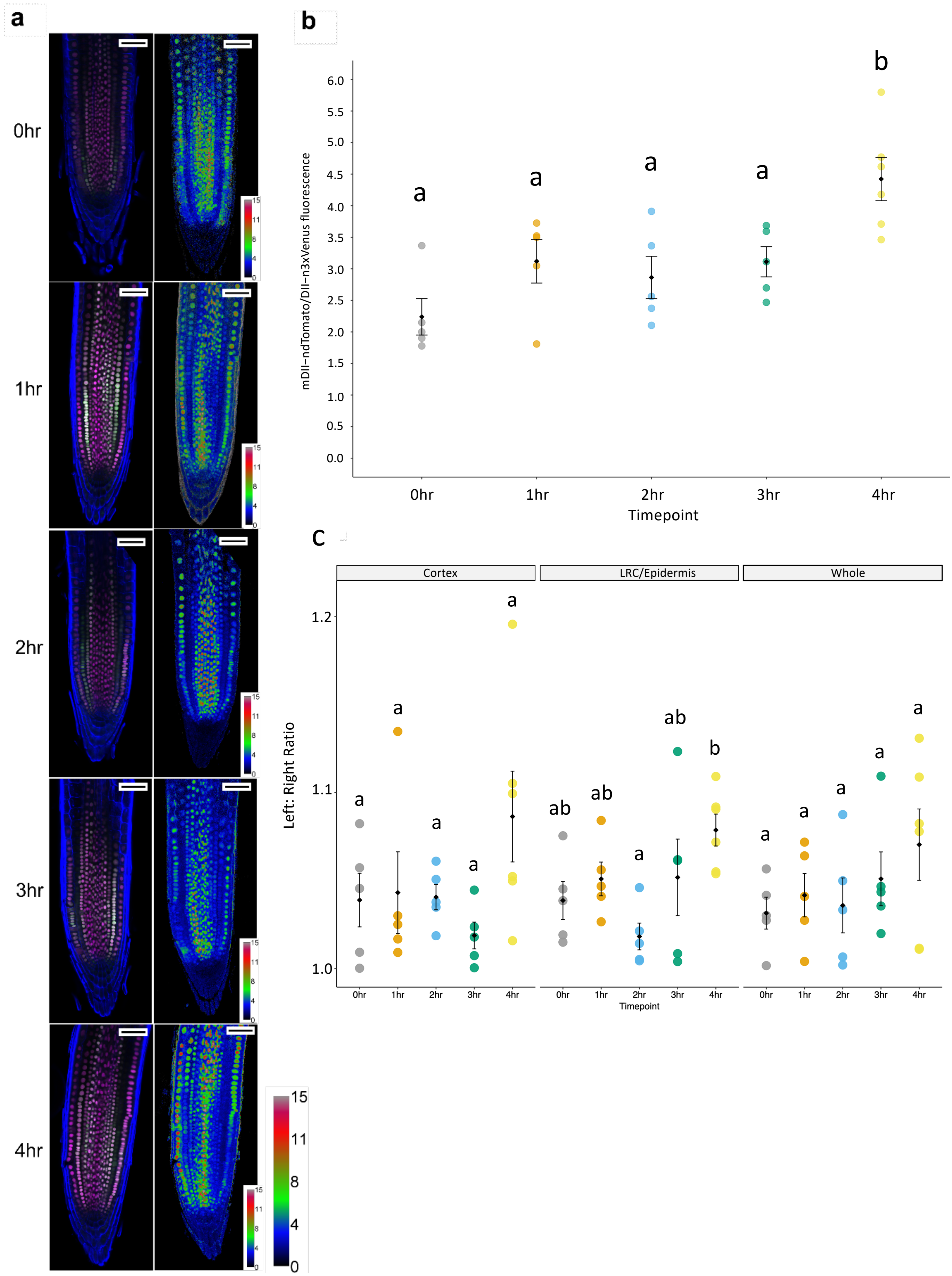

Figure S15. Confocal imaging of R2D2 in roots responding to a barrier between 0-4 h. Barriers were placed in front of root tips 6 DAS. a) R2D2 fluorescence at the root tip at 0-4 hours with accompanying ratio metric image of generated from mDII-ndTomato/ DII-m3xVenus fluorescence using ImageJ. For ratiometric images calibration bar indicates mean grey value. b) Ratio of mDII-ndTomato/ DII-m3xVenus fluorescence. c) Ratio of auxin level across the left and right sides of the root tip. Scale bar = 50 µm. Black circles and error bars represent mean ± SE. Coloured circles represent distribution of individual data points. Letters indicate significant difference after post-hoc TUKEY test, $P < 0.05$.

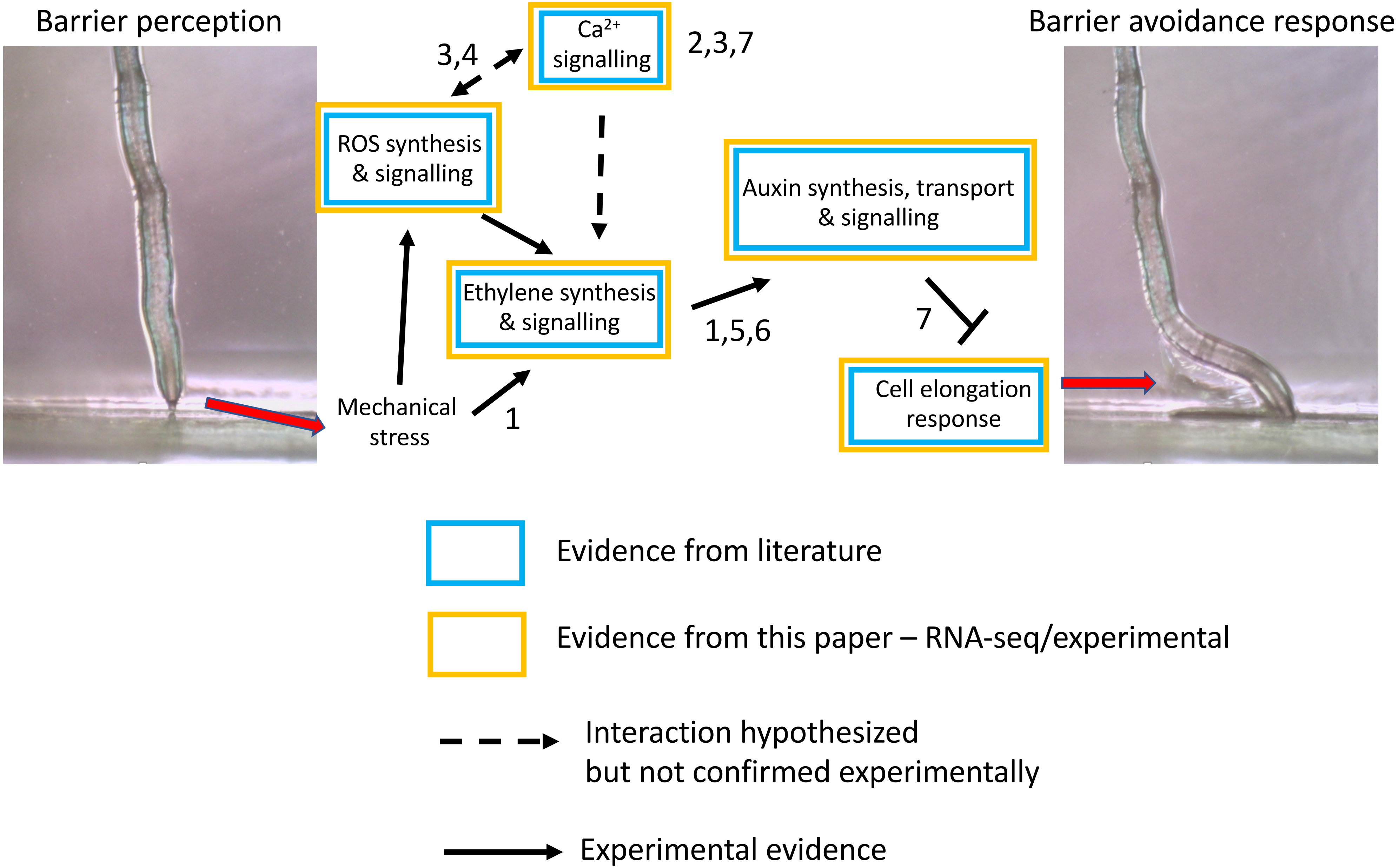
Barrier perception
Barrier avoidance response
Ca2+ signalling
2,3,7
3,4
ROS synthesis & signalling
Auxin synthesis, transport & signalling
Ethylene synthesis & signalling
1,5,6
7
Cell elongation response
Mechanical stress
1
Evidence from literature
Evidence from this paper – RNA-seq/experimental
Interaction hypothesized but not confirmed experimentally
Experimental evidence

Fig. S16

Fig. S16. Pathways involved in the root barrier response. Hypotheses generated from results presented throughout the thesis and from previously published literature (numbers). Arrows show hypothesised positive interaction/relationship; t-bars inhibition or negative relationship. Boxed are colour-coded according to the source of evidence. Numbers refer to published literature 1. Okamoto *et al*. (2008); Okamoto & Takahashi (2019) 2. Shih *et al*. (2014) 3. Monshausen *et al*. (2009) 4. Gilroy *et al*. (2014, 2016) 5. Růžička *et al*. (2007) 6. Strader *et al*. (2010) 7. Lee *et al*. (2020).

Table S1 Primers used for qRT-PCR.

Locus ID	Gene Name/Symbol	Forward Primer 5'-3'	Reverse Primer 5'-3'
AT4G05320	UBQ10	GGCCTTGTATAATCCCTGATGAATAAG	AAAGAGATAACAGGAACGGAAACATAGT
AT5G25760	PEX4	CTGCGACTCAGGGAATCTTTCTAA	TTGTGCCATTGAATTGAACCC
ATMG00560	RPL2	AAGCTCTCCGGGGTTTGAGG	GGATGGTCATTCTGGGCGGA
AT1G77840	IF2/IF5	TCTTACAAGGAAGTTGGGCAGT	TCTTACAAGGAAGTTGGGCAGT
AT4G14560	IAA1	ACAATCCCAAGAAGAGCAATAAC	CTCACTATACTTTAACGGAGAAG
AT3G23030	IAA2	CCTCCTACCAAAACTCAAATCGTT	CGTAGCTCACACTGTTGTTGTTCT
AT3G23240	ERF1	GGTATTAGGGTTTGGCTCGG	CCGAAAGCGACTCTTGAACT

Table S2 NADPH-oxidase genes identified through RNA-Seq that are upregulated during the barrier response at 6 hours.

Locus ID	Primary Gene Symbol	Log FC 6hr	Gene Description
AT1G09090	RBOHB	0.608	NADPH-oxidase AtrbohB plays a role in seed after-ripening. Major producer of superoxide in germinating seeds. AtrbohB pre-mRNA is alternatively spliced in seeds in a hormonally and developmentally regulated manner.
AT1G64060	RBOHF	0.608	Interacts with AtrbohD gene to fine tune the spatial control of ROI production and hypersensitive response to cell in and around infection site.
AT4G11230	RBOHI	0.694	NADPH-oxidase RbohI is expressed highly in seeds and roots. Mutants have inreased sensitivity to osmotic stress suggesting a role in mediating cellular response to stress in roots.
AT5G07390	RBOHA	1.063	respiratory burst oxidase homolog A
AT5G47910	RBOHD	0.743	NADPH/respiratory burst oxidase protein D (RbohD).Interacts with AtrbohF gene to fine tune the spatial control of ROI production and hypersensitive response to cell in and around infection site.
AT5G51060	RHD2	1.145	RHD2 (along with RHD3 and RHD4) is required for normal root hair elongation. Has NADPH oxidase activity. Gene is expressed in the elongation and differention zone in trichoblasts and elongating root hairs. Required for the production of reactive oxygen species in response to extracellular ATP stimulus. The increase in ROS production stimulates Ca^{2+} influx.

Table S3 List of genes that act as reactive oxygen species (ROS) scavengers identified in the RNA-Seq data of differentially expressed genes in response to a barrier log2fc identified with P-value <0.05.

**Locus ID**	**Gene Name/Symbol**	**Enzyme Family**	**LogFC**
AT1G01580	FERRIC REDUCTION OXIDASE 2 (FRO2)	NADPH oxidase-like	1.918
AT1G03850	GLUTAREDOXIN 13 (GRXS13)	Glutaredoxin (GLR)	0.970
AT1G09090	RESPIRATORY BURST OXIDASE HOMOLOG B (RBOHB)	NADHP oxidase	0.608
AT1G19230	(ATRBOHE)	NADPH oxidase	0.977
AT1G23020	FERRIC REDUCTION OXIDASE 3 (FRO3)	NADPH oxidase	0.849
AT1G28480	(GRX480)	Glutaredoxin (GLR)	1.931
AT1G45145	THIOREDOXIN H-TYPE 5 (TRX5)	Thioredoxins (Trx)	0.760
AT1G60740		Peroxiredoxin (PrxR)	0.883
AT1G64060	RESPIRATORY BURST OXIDASE PROTEIN F (RBOH F)	NADPH oxidase	0.608
AT2G31570	GLUTATHIONE PEROXIDASE 2 (GPX2)	Glutathione Peroxidase (GPX)	0.804
AT3G27820	MONODEHYDROASCORBATE REDUCTASE 4 (MDAR4)	Monodehydroascorbate Reductase (MDAR)	0.523
AT4G11230	(RBOHI)	NADPH oxidase	0.694
AT4G25090	(ATRBOHG)	NADPH oxidase	1.595
AT4G35970	ASCORBATE PEROXIDASE 5 (APX5)	Ascorbate Peroxidase (APX)	0.855
AT5G07390	RESPIRATORY BURST OXIDASE HOMOLOG A (RBOHA)	NADPH oxidase	1.063
AT5G23980	FERRIC REDUCTION OXIDASE 4 (FRO4)	NADPH oxidase-like	1.680
AT5G23990	FERRIC REDUCTION OXIDASE 5 (FRO5)	NADPH oxidase-like	2.451
AT5G36270		Dehydroascorbate Reductase (DHAR)	2.578
AT5G47910	RESPIRATORY BURST OXIDASE HOMOLOGUE D (RBOHD)	NADPH oxidase	0.743
AT5G51060	ROOT HAIR DEFECTIVE (RHD2)	NADPH oxidase	1.145
AT3G62930	GRXS6	Glutaredoxin (GLR)	-0.658

Supporting Information references

Gilroy S, Białasek M, Suzuki N, Górecka M, Devireddy AR, Karpińsk S., Mittler R. 2016. ROS, calcium, and electric signals: Key mediators of rapid systemic signaling in plants. *Plant Physiology* **171**: 1606–1615.

Gilroy S, Suzuki N, Miller G, Choi WG, Toyota M, Devireddy AR, Mittler R. 2014. A tidal wave of signals: Calcium and ROS at the forefront of rapid systemic signaling. *Trends in Plant Science* **19**: 623–630.

Lee HJ, Kim HS, Park JM, Cho HS, Jeon JH. 2020. PIN-mediated polar auxin transport facilitates root obstacle avoidance. *New Phytologist* **225**: 1285–1296.

Monshausen GB, Bibikova TN, Weisenseel MH, Gilroy S. 2009. Ca^{2+} regulates reactive oxygen species production and pH during mechanosensing in arabidopsis roots. *Plant Cell* **21**: 2341–2356.

Okamoto T, Takahashi, T. 2019. Ethylene signaling plays a pivotal role in mechanical-stress-induced root-growth cessation in *Arabidopsis thaliana*. *Plant Signaling and Behavior* **14**: 1669417.

Okamoto T, Tsurumi S, Shibasaki K, Obana Y, Takaji H, Oono Y, Rahman A. 2008. Genetic dissection of hormonal responses in the roots of arabidopsis grown under continuous mechanical impedance. *Plant Physiology* **146**: 1651–1662.

Růžička K, Ljung K, Vanneste S, Podhorská R, Beeckman T, Friml J, Benková E. 2007. Ethylene regulates root growth through effects on auxin biosynthesis and transport-dependent auxin distribution. *Plant Cell* **19**: 2197– 2212.

Shih HW, Miller ND, Dai C, Spalding EP, Monshausen GB. 2014. The receptor-like kinase FERONIA is required for mechanical signal transduction in Arabidopsis seedlings. *Current Biology* **24**: 1887–1892.

Strader LC, Chen GL, Bartel B. 2010. Ethylene directs auxin to control root cell expansion. *Plant Journal* **64**: 874–884.

Supek F, Bošnjak M, Škunca N, Šmuc T. 2011. Revigo summarizes and visualizes long lists of gene ontology terms. *PLoS ONE* **6**: e21800.
